# Marijuana Is Associated With a Hormonal Imbalance Among Several Habits Related to Male Infertility: A Retrospective Study

**DOI:** 10.3389/frph.2022.820451

**Published:** 2022-02-17

**Authors:** Thiago A. Teixeira, Ivan Iori, Gustavo Andrade, Paulo H. N. Saldiva, Joël R. Drevet, Elaine M. F. Costa, Jorge Hallak

**Affiliations:** ^1^Androscience—Science and Innovation Center in Andrology and High-Complex Clinical and Research Andrology Laboratory, São Paulo, Brazil; ^2^Division of Urology, Hospital das Clinicas, University of São Paulo Medical School, São Paulo, Brazil; ^3^Institute of Advanced Studies, University of São Paulo, São Paulo, Brazil; ^4^Faculty of Medical Sciences, State University of Campinas (UNICAMP), Campinas, Brazil; ^5^Reproductive Toxicology Unit, Department of Pathology, University of São Paulo Medical School, São Paulo, Brazil; ^6^GReD Institute, CRBC Building, Faculty of Medicine, CNRS-INSERM-Université Clermont Auvergne, Clermont-Ferrand, France; ^7^Division of Endocrinology, Hospital das Clinicas, University of São Paulo Medical School, São Paulo, Brazil

**Keywords:** marijuana, male infertility, sedentary lifestyle, tobacco smoking, estradiol

## Abstract

Marijuana is one of the most consumed drugs worldwide. There is increasing evidence of an association between marijuana and male infertility. This study intends to assess the repercussion of marijuana smoking and other habits (sedentary lifestyle, alcohol, and tobacco use) in the testicular function of infertile men seeking andrological evaluation. A retrospective study was performed using medical records data of men aged 18–59 years from 2009 to 2017. Complete semen analyses, sperm functional tests, SHBG, and hormonal levels, testosterone-to-estradiol ratio (T/E_2_), and testis volume were evaluated. Exclusion criteria included cryptorchidism, infertility caused by genetic or infectious diseases, and cancer. A multiple linear regression analysis was performed to investigate which habit could predict certain parameters using the software SPSS 23.0 (*P* < 0.05). In a sample of 153 men, semen parameters, testosterone levels, and testis volume were not significantly influenced. Marijuana use had the broader hormonal changes since it influences estradiol (*P* = 0.000; *B* = −11.616), prolactin (*P* = 0.000; *B* = 3.211), SHBG levels (*P* = 0.017; *B* = 7.489), and T/E_2_ (*P* = 0.004; *B* = 14.030). Sedentary lifestyle (*P* = 0.028; *B* = 1.279) and tobacco smoking (*P* = 0.031; *B* = −2.401) influenced the prolactin levels. Marijuana is associated with hormonal imbalance in this infertile cohort by lowering estradiol levels and inhibiting aromatase function.

## Introduction

Produced on every continent, marijuana (cannabis made from the dried flowers and leaves of the plant *Cannabis sativa*) is one of the most widely consumed drugs with ~188 million users or 3.8% of the world's population between 15 and 64 years of age (1). Although marijuana is illegal in most countries, its use is currently rising worldwide, mainly for recreational use and recently for alleged and realistic medical purposes, particularly among men of childbearing age (2).

Infertility is a disease described as the inability to conceive after 12 months of regular sexual intercourse without using a contraceptive method (3). The male reproductive function has been the subject of particular attention in recent years due to the accumulation of data on a possible deterioration in sperm counts and quality related to various environmental and behavioral factors (4). In recent decades, numerous investigations have raised the impacts of lifestyle and environment on male fertility (5). Among the wide range of environmental factors that can affect men's fertility, some evidence suggests an association between chronic marijuana use and male infertility (6).

Overall, most human studies associate marijuana use with a deleterious impact on male fertility status, mainly because of the correlation with lower sperm concentrations, morphologic abnormalities, and reduced motility and viability (6–9). Nonetheless, current evidence seems conflicting regarding the effects of cannabis on male reproductive endocrine function, with an emphasis on the inconclusive findings in testosterone levels, concomitant with reduced luteinizing hormone (LH) and unchanged follicle-stimulating hormone (FSH) concentrations (7–12).

The study reported here aims to evaluate the effects of marijuana, within other conditions (obesity, sedentary lifestyle, smoking, alcohol consumption), on the spermatozoa function and sex hormone levels in reproductive failure situations in patients attending an andrology setting.

## Materials and Methods

### Study Design and Patients

This cross-sectional study was carried out using data from the medical records of 316 infertile men aged 18–60 years who attended a referral center for male sexual and reproductive health in São Paulo, Brazil (Androscience, Science & Innovation Center, High Complex Clinical & Research Andrology Laboratory) between 2009 and 2017. All men presented two complete seminal analyses (13) and were assessed for total serum testosterone concentration collected in the initial clinical assessment (maximum interval of 15 days). The assessment also included a mandatory measurement of testicular volume (TV) using an ultrasound with calculated TV deduced from the ellipsoidal formula:


$$\begin{array}{l} \text{TV }\left(\text{c}{\text{m}}^{3}\right)=\left[\text{length }\left(\text{cm}\right)\text{ X width }\left(\text{cm}\right)\text{ X depth }\left(\text{cm}\right)\text{ X }0.52\right] \end{array}$$

**Figure 1:**
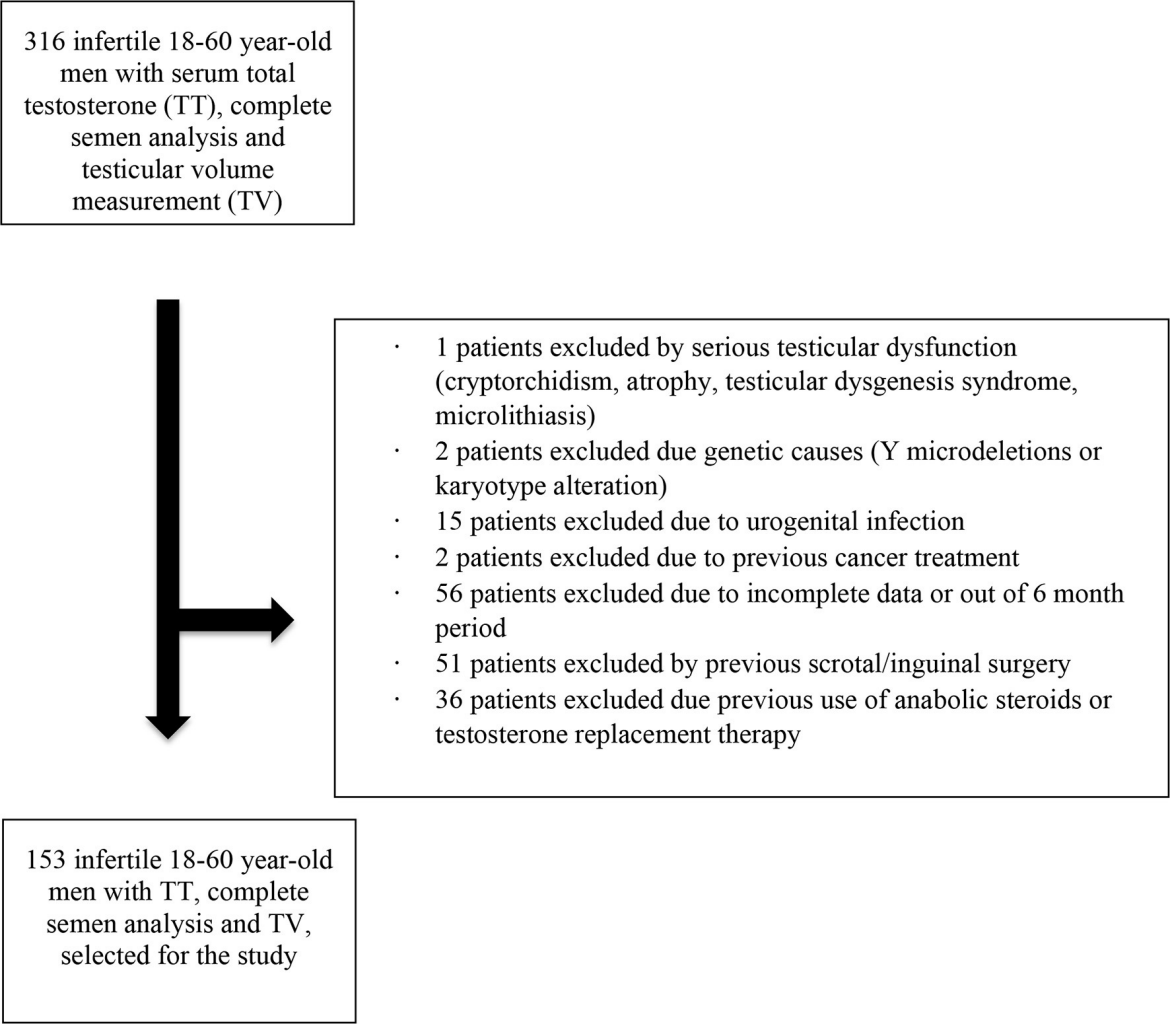
Patient selection diagram.

Exclusion criteria included cryptorchidism, or other severe testicular dysfunction (atrophy, testicular dysgenesis syndrome), genetic infertility (Y-chromosome micro-deletions, karyotype abnormalities), clinical infection of the urogenital tract, previous cancer treatments (radiation or chemotherapy), previous scrotal surgeries, and use of anabolic steroids or testosterone replacement therapy. As a result, of the 316 patients, the final sample size was reduced to 153 men (Figure 1). Varicocele, which accounted for a large proportion of cases, was not considered as an exclusion criterium. It was assessed and classified accordingly to Dubin and Amelar's criteria (14). It was included in the regression model to control its influence, as was the case with patients clinically classified as obese.

Our assessment also included the patient's information regarding obesity status, sedentary lifestyle, the extent of smoking, alcohol, and marijuana use. The obesity status was assessed using the body mass index (BMI), and two sub-groups of patients were defined. Patients with a BMI <30 have been considered “non-obese,” while patients with a BMI >30 have been considered obese (15). The extent of tobacco smoking was categorized as less than ten pack-years (moderate smoker), equivalent or more than ten pack-years (heavy smoker). “Pack-years” classically corresponds to a person smoking one pack of cigarettes per day for less or more than 10 years (16). The daily consumption of alcoholic beverages was divided into two groups (< three doses/day and ≥ three doses/day), according to WHO standards of presumed damage, in which one dose corresponds to 40 ml of whisky or a 140 ml glass of wine or a 330 ml can of beer (17). Marijuana smoking was defined as drug use at least once a week for a minimum of 12 months (18).

The study was conducted according to the guidelines of the Declaration of Helsinki (19), and approved by the Institutional Review Board (or Ethics Committee) of the Faculty of Medicine of the University of São Paulo, Brazil (registration number: CAAE 92728018.0.0000.0068). The Institutional Ethics Committee exempted using an informed consent form because all data were collected from medical records, and there was no clinical intervention.

### Seminal and Hormonal Analysis

All semen analyses were performed in Androscience, Science and Innovation Center, High-Complex Clinical and Research Andrology Laboratory. Samples were obtained by masturbation after sexual abstinence for 2–5 days. Semen analysis was performed according to WHO guidelines (13). It included the following parameters: pH, volume (ml), concentration (×10^6^/ml), progressive motility (%) and total motility (%), total sperm count (×10^6^), total motile sperm count (×10^6^), total sperm with progressive motility (×10^6^). Sperm morphological analysis was performed using the strict criteria (20). For the statistical model, the mean value for each semen parameter was calculated using the values from the two baseline semen analysis, assuring that no intervention was performed between these two semen collections.

Data from biochemical markers of sperm function and sperm function assays were also collected. Briefly, anti-sperm antibodies were evaluated using the commercial Marscreen kit (Bioscreen New York, NY, USA). According to Huszar et al. (21), creatine kinase activity was evaluated using a Spectroquant Pharo 300 spectrophotometer (Marck, Germany). The results are expressed in units for 10^8^ spermatozoa. The levels of reactive oxygen species (ROS) were evaluated by chemiluminescence (Autolumat Plus luminometer, Bertold Technologies, Germany), and the results were expressed in 10^4^ photons/minute (cpm) for 20 × 10^6^ spermatozoa. The assessment of sperm DNA integrity was performed by the SCSA® (Sperm Chromatin Structure Assay) method, and data were analyzed to determine the DNA Fragmentation Index (DFI%).

Levels of LH (in μU/l), FSH (in μU/l), total testosterone (TT, in ng/dl), prolactin (PRL, in ng/ml), estradiol (E2, in pg/ml), and sex hormone-binding globulin (SHBG, in nmol/l) were measured by an electrochemiluminescent immunoassay kit (ECLIA kit; Roche Diagnostics, Mannheim, Germany). Hormones and SHBG measurements were extracted from a specific database and performed in the same laboratory following the morning sampling recommendation.

### Statistical Analysis

Patients were characterized by age, obesity, the presence of clinical varicocele (14), personal information about tobacco and cannabis use, alcohol consumption, and sedentary lifestyle. Semen parameters, hormonal levels (LH, FSH, total testosterone, prolactin, estradiol), SHBG content, and testosterone/estradiol ratio (T/E2) were recorded and evaluated as variables. The categorical data were described by absolute number, frequency, and proportion. Constantly changing data were described by the mean and standard deviation (SD). The data were subjected to the Shapiro-Wilk test to confirm the distribution of normality (*P* < 0.01). Multiple linear regression analysis was performed to determine which habit or lifestyle factors (tobacco use, daily alcohol consumption, marijuana use, and sedentary lifestyle) could influence specific sperm parameters or hormone levels. During the analysis, age (≥45 years) and clinical varicocele were included as predictors. The ANOVA test was used to verify the association between semen or hormonal parameters and other clinical or epidemiological characteristics. All statistical analyses were performed using SPSS 23.0 (IBM Corp, Armonk, NY, USA) with a significance level of *P* < 0.05.

## Results

Table 1 presents all the characteristics of the cohort being evaluated (*n* = 153 infertile males). Note the following: mean age of 38.05 ± 8.81 years (IC: 37.01–39.08); about half of the cohort was overweight, and 27 patients (17.65%) were classified as obese; 95 subjects presented a clinical varicocele, which represents a high proportion (62.09%) of the cohort but is typical of the infertile cases encountered in a referral male infertility center. Mean testicular volume was around 16 ml on both sides.

**Table 1:** Baseline clinical and laboratorial characteristics of infertile patients (*n* = 153), 2009–2017.

**Clinical or laboratorial feature**	**Values**
Age (years), mean ± SD (95% IC)	38.05 ± 8.81 (37.01–39.08)
*BMI* (kg/m^2^), mean ± SD (95% IC)^a^	27.02 ± 3.86 (26.52–27.52)
Underweight, *n* (%)	1 (0.65)
Normal BMI, *n* (%)	50 (32.68)
Overweight, *n* (%)	75 (49.02)
Obese, *n* (%)	27 (17.65)
Presence of clinical varicocele, *n* (%)	95 (62.09)
Right, *n* (%)	1 (0.65)
Left, *n* (%)	26 (16.99)
Bilateral, *n* (%)	68 (44.44)
No varicocele, *n* (%)	58 (37.91)
**Testicular volume–ultrasound (ml)**	
Right testis, mean ± SD (95% IC)	16.57 ± 5.23 (15.40–16.79)
Left testis, mean ± SD (95% IC)	16.10 ± 5.31 (15.89–17.26)
**Tobacco smoking**	
Current, *n* (%) all ≥10 year-pack	7 (4.57)
Ex-smoker, *n* (%) all ≥10 year-pack	8 (5.23)
Never, *n* (%)	138 (90.19)
**Sedentary lifestyle**	
Yes, *n* (%)	74 (48.37)
No, *n* (%)	79 (51.63)
**Daily alcohol consumption**	
No or <3 doses/day, *n* (%)	98 (64.05)
≥3 doses/day, *n* (%)	55 (35.94)
**Distilled drink (whiskey)**	
Time (year), mean ± SD (95% IC)	15.33 ± 9.16 (12.36–18.30)
Volume (ml/day), mean ± SD (95% IC)	13.77 ± 11.35 (9.93–17.61)
**Fermented drink (beer)**	
Time (year), mean ± SD (95% IC)	14.47 ± 7.60 (12.38–16.57)
Volume (ml/day), mean ± SD (95% IC)	158.66 ± 112.77 (128.17–189.14)
**Fermented drink (wine)**	
Time (year), mean ± SD (95% IC)	11.38 ± 8.38 (8.58–14.17)
Volume (ml/day), mean ± SD (95% IC)	75.07 ± 53.54 (60.16–89.97)
**Marijuana smoking (≥ 1x/week/12 months)**	
Yes, *n* (%)	42 (27.45)
No, *n* (%)	111 (72.55)
**Seminal parameters, mean ± SD (range)** ^**b**^ ^,^ ^**c**^	
pH	8.04 ± 0.29 (7.00–9.00)
Semen volume (ml)	3.01 ± 1.70 (0.25–10.00)
Sperm concentration (10^6^/ml)	72.59 ± 91.26 (0.00–800.00)
Total number of spermatozoa (10^6^)	189.98 ± 216.31(0.00–1,440.00)
Total number of motile spermatozoa (10^6^)	124.05 ± 155.25 (0.00–1,152.00)
Total number of spermatozoa with progressive motility (10^6^)	66.33 ± 99.99 (0.00–748.00)
Total motility (%)	53.01 ± 24.64 (0.00–90.00)
Total progressive motility (%)	32.46 ± 21.39 (0.00–75.00)
Kruger normal morphology (%)	2.32 ± 2.67 (0.00–13.00)
**Semen functional tests, mean ± SD (range)**	
Creatine-kinase activity (U/10^8^ sperm)	2.39 ± 10.75 (0.00–103.62)
Anti-sperm antibodies + (%)	20.19 ± 13.74 (1.00–66.00)
ROS levels (10^4^ cpm/20 × 10^6^)	7.20 ± 19.53 (0.00–137.50)
Sperm DNA fragmentation index (%)	42.21 ± 21.89 (5.00–90.00)
**Hormonal parameters, mean ± SD (range)**	
LH (μU/L)	4.31 ± 2.51 (0.06–19.20)
FSH (μU/L)	5.23 ± 5.18 (0.22–41.50)
Total testosterone (ng/dl)	495.47 ± 188.33 (164.00–1,231.00)
Prolactin (ng/ml)	8.33 ± 4.34 (1.40–31.00)
Estradiol (pg/ml)	25.46 ± 12.49 (1.00–62.00)
SHBG (nmol/l)	35.36 ± 35.35 (6.70–415.00)

a *According to WHO Guidelines (2010) (9)*.

b *According to WHO Guidelines (2010) (7)*.

c *According to Kruger et al. (12)*.

*SD, standard deviation; 95% IC, 95% interval confidence; BMI, body mass index; WHO, World Health Organization; ROS, reactive oxygen species; DNA, deoxyribonucleic acid; LH, luteinizing hormone; FSH, follicle-stimulating hormone; SHBG, sex hormone-binding globulin*.

Data represent the mean basic semen parameters of the infertile population in the study (i.e., semen volume, sperm count, sperm concentrations, percentage of progressive motility, percentage of normal morphology) were on average, i.e., above the 5% minimum percentile considered acceptable, according to WHO 2010 recommendations (7). Table 2 illustrates the seminal alterations observed in the infertile cohort analyzed according to WHO (2010) recommendations (13). Regarding lifestyle factors, tobacco smoking affected <10% of the cohort, and almost half of the patients were classified as having a sedentary lifestyle (48.37%, *n* = 74). Heavy to moderate alcohol consumption affected about one-third of patients (35.94%; *n* = 55). Finally, marijuana use concerned 27.45% of patients (*n* = 42). It is essential to mention that none of the cannabis smokers use other commonly associated (non-prescribed) drugs, except for two out of 42 patients, who were tobacco and marijuana smokers.

**Table 2:** Basic semen abnormalities found in the infertile cohort (*n* = 153), 2009–2017.

**Seminal parameter**	**Alteration**	**Frequency**	**%**
pH	Normal^#^	151	98.69
	Acid (<7, 2)	2	1.31
Semen volume (mL)	Normal^#^	136	88.88
	Decreased (<1, 5)	17	11.12
Sperm concentration (10^6^/mL)	Norm^#^	117	76.47
	Oligozoospermia	36	23.53
Total number of spermatozoa (10^6^)	Normal^#^	114	74.50
	Decreased	39	25.50
Total motility (%)	Normal^#^	119	77.77
	Decreased	34	22.23
Total progressive motility (%)	Normal^#^	77	50.32
	Asthenozoospermia	76	49.68
Kruger normal morphology (%)	Normal	31	20.26
	Teratozoospermia	111	72.54
	NR	11	7.20

*NR, not reported in the medical record; WHO, World Health Organization*.

# *The 5% percentile was set as the lower reference values for normality, according to WHO Guidelines (2010) (7)*.

After testing the linearity assumptions, normal distribution, and non-correlation errors, multiple linear regression analyses demonstrated that only serum estradiol (*p* < 0.01) and prolactin concentrations (*p* < 0.01), SHBG content (*p* = 0.01), T/E2 ratio (*p* = 0.01), and to a lesser extent, semen volume (*p* = 0.02) appeared to be influenced by the lifestyle parameters (Table 3). None of the other basic seminal parameters, sperm functional tests, and other serum hormones examined were significantly affected (Table 3). It is worth noting the elevated means for all sperm functional tests, mainly the DNA fragmentation index (42.21 ± 21.89 %) and the ROS levels (7.20 ± 19.53 × 10^4^ cpm/20 × 10^6^), far beyond the considered “normal” values (22, 23).

**Table 3:** Summary of the multiple linear regression analysis model with constant predictors^*^ for hormonal/seminal parameters and testicular volume.

**Parameter**	***R***	***R* ^2^**	***Adjusted R* ^2^**	***P***
Estradiol	0.44	0.19	0.15	**<0.001**
Prolactin	0.40	0.16	0.12	**<0.001**
Total testosterone	0.22	0.05	0.00	0.36
LH	0.24	0.06	0.01	0.29
FSH	0.27	0.07	0.02	0.16
T/E_2_	0.35	0.12	0.08	**0.01**
SHBG	0.34	0.12	0.07	**0.01**
pH	0.18	0.03	−0.01	0.70
Semen volume	0.34	0.11	0.07	**0.02**
Sperm concentration	0.29	0.08	0.04	0.08
Total number of spermatozoa	0.27	0.07	0.02	0.16
Total number of motile spermatozoa	0.30	0.09	0.04	0.06
Total number of spermatozoa with progressive motility	0.23	0.05	0.01	0.32
Total motility	0.21	0.05	0.00	0.41
Total progressive motility	0.20	0.04	−0.01	0.53
Kruger normal morphology	0.25	0.06	0.01	0.30
Creatine-kinase activity	0.28	0.08	−0.03	0.67
Anti-sperm antibodies +	0.29	0.08	0.00	0.40
ROS levels	0.49	0.24	0.11	0.12
Sperm DNA fragmentation index	0.23	0.05	−0.14	0.96
Right testis (orquidometer)	0.29	0.08	0.03	0.16
Left testis (orquidometer)	0.30	0.09	0.04	0.12
Right testis (ultrasound)	0.18	0.03	−0.01	0.67
Left testis (ultrasound)	0.11	0.01	−0.03	0.47

*ANOVA test, P < 0.05*.

* *Model predictors: (1) Age ≥ 45 years, Obesity [BMI ≥ 30 kg/m^2^], (3) Sedentary lifestyle, (4) current tobacco smoking ≥ 10 year-pack, (5) daily alcohol consumption ≥ 3 doses according to WHO (10), (6) marijuana smoker [≥ 1× per week/1 year], (7) presence of clinical varicocele*.

*LH, luteinizing hormone; FSH, follicle-stimulating hormone; T/E_2_, Testosterone-estradiol ratio; SHBG, sex hormone-binding globulin; WHO, World Health Organization; ROS, reactive oxygen species; DNA, deoxyribonucleic acid; BMI, body mass index. Bold values mean P < 0.05*.

The beta and B regression coefficients (standardized and unstandardized coefficients, respectively), determined by the multiple linear regression analysis, were used to rank the predictors that had the most significant effect on the outcome variable (Tables 4–8). Among the distinct lifestyle predictors analyzed (obesity, physical inactivity, smoking, alcohol use, marijuana use), marijuana use revealed to provoke the most substantial effect on serum estradiol (Table 4: ß = −0.35, p < 0.01) and prolactin levels (Table 5: ß = 0.30; p < 0.01). Interestingly, marijuana use was inversely correlated with serum estradiol while positively correlated with serum prolactin. Moreover, serum prolactin levels were also negatively influenced by smoking and obesity, whereas they were positively influenced by a sedentary lifestyle (Table 5). Confirming in part the above observation, and in the absence of any observed effect on serum testosterone levels, marijuana use was also responsible for the most potent positive effect on the T/E2 ratio (Table 6: ß = 0.24; p < 0.01). Regarding the variable “SHBG content,” marijuana use also had the most influential effect (Table 7: ß = 0.19; p = 0.02). Finally, while it appeared in the Table 3 that semen volume could be correlated with the constant predictors evaluated, the Table 8 demonstrates that this parameter is not significantly influenced by marijuana use (ß = −0.03; p = 0.67). Unsurprisingly, age significantly influenced each of the dependent variables analyzed, except for the T/E_2_ ratio (Table 6: ß = 0.1; p = 0.2).

**Table 4:** Coefficients of the multiple linear regression analysis model for dependent variable Estradiol.

Model predictor^*^	**Unstandardized coefficients**		**Standardized coefficient**	**P**	**95% Confidence interval for beta**	
	**B**	**Stand error**	**Beta**		**Lower bound**	**Upper bound**
Estradiol	**38.42**	4.27	-	**0.00**	29.97	46.87
Age	**−0.27**	0.10	−0.21	**0.01**	−0.47	−0.08
Obesity	−1.23	2.12	−0.04	0.56	−5.43	2.96
Sedentary lifestyle	2.05	1.71	0.01	0.23	−1.32	5.42
Tobacco smoking	2.57	3.34	0.06	0.44	−4.03	9.17
Alcohol consumption	−0.04	1.76	−0.00	0.99	−3.53	3.44
Marijuana use	**−11.61**	2.55	−0.35	**0.00**	−16.66	−6.56
Varicocele	−2.14	1.86	−0.09	0.25	−5.82	1.54

*ANOVA test, P < 0.05*.

* *Model Predictors: (1) Age ≥ 45 years, (2) Obesity [BMI ≥ 30 kg/m^2^], (3) Sedentary lifestyle, (4) Current tobacco smoking ≥ 10 year-pack, (5) Daily alcohol consumption ≥ 3 doses according to WHO (10), (6) Marijuana smoker [≥ 1× per week/1 year], (7) Presence of clinical varicocele; BMI, body mass index; WHO, World Health Organization. Bold values mean P < 0.05*.

**Table 5:** Coefficients of the multiple linear regression analysis model for dependent variable Prolactin.

Model predictor^*^	**Unstandardized coefficients**		**Standardized coefficient**	***P***	**95% Confidence interval for beta**	
	**B**	**Stand error**	**Beta**		**Lower bound**	**Upper bound**
Prolactin	**10.99**	1.43	-	**0.00**	8.16	13.82
Age	**−0.08**	0.03	−0.18	**0.02**	−0.14	−0.01
Obesity	−0.21	0.70	−0.02	0.76	−1.61	1.18
Sedentary lifestyle	**1.28**	0.58	0.18	**0.03**	0.14	2.42
Tobacco smoking	**−2.40**	1.10	−0.17	**0.03**	−4.58	−0.22
Alcohol consumption	−0.10	0.59	−0.01	0.87	−1.28	1.08
Marijuana use	**3.21**	0.84	0.30	**0.00**	1.54	4.88
Varicocele	−0.83	0.63	−0.10	0.19	−2.08	0.42

*ANOVA test, P < 0.05*.

* *Model Predictors: (1) Age ≥ 45 years, (2) Obesity [BMI ≥ 30 kg/m^2^], (3) Sedentary lifestyle, (4) Current tobacco smoking ≥ 10 year-pack, (5) Daily alcohol consumption ≥ 3 doses according to WHO (10), (6) Marijuana smoker [≥ 1× per week/1 year], (7) Presence of clinical varicocele; BMI, body mass index; WHO, World Health Organization. Bold values mean P < 0.05*.

**Table 6:** Coefficients of the multiple linear regression analysis model for dependent variable *T/E*_2_.

Model predictor^*^	**Unstandardized coefficients**		**Standardized coefficient**	***P***	**95% Confidence interval for beta**	
	**B**	**Stand error**	**Beta**		**Lower bound**	**Upper bound**
T/E_2_	10.40	7.13	-	0.15	−3.69	24.49
Age	0.21	0.16	0.10	0.20	−0.11	0.54
Obesity	−3.69	3.41	−0.09	0.28	−10.44	3.05
Sedentary lifestyle	−2.42	2.73	−0.07	0.37	−7.82	2.97
Tobacco smoking	6.26	5.38	0.09	0.25	−4.39	16.90
Alcohol consumption	4.18	2.87	0.11	0.15	−1.49	9.85
Marijuana use	**14.03**	4.80	0.24	**0.00**	4.55	23.51
Varicocele	2.81	3.02	0.07	0.35	−3.17	8.79

*ANOVA test, P < 0.05*.

* *Model Predictors: (1) Age ≥ 45 years, (2) Obesity [BMI ≥ 30 kg/m^2^], (3) Sedentary lifestyle, (4) Current tobacco smoking ≥ 10 year-pack, (5) Daily alcohol consumption ≥ 3 doses according to WHO (10), (6) Marijuana smoker [≥ 1× per week / 1 year], (7) Presence of clinical varicocele; T/E_2_, Testosterone-estradiol ratio; BMI, body mass index; WHO, World Health Organization. Bold values mean P < 0.05*.

**Table 7:** Coefficients of the multiple linear regression analysis model for dependent variable SHBG.

Model predictor^*^	**Unstandardized coefficients**		**Standardized coefficient**	***P***	**95% confidence interval for beta**	
	**B**	**Stand error**	**Beta**		**Lower bound**	**Upper bound**
SHBG	**16.98**	5.15	-	**0.00**	6.81	27.16
Age	**0.40**	0.12	0.26	**0.00**	0.16	0.64
Obesity	−3.89	2.53	−0.12	0.13	−8.89	1.11
Sedentary lifestyle	2.97	2.07	0.12	0.15	−1.12	7.07
Tobacco smoking	−1.36	4.06	−0.03	0.74	−9.39	6.66
Alcohol consumption	1.48	2.15	0.05	0.49	−2.77	5.74
Marijuana use	**7.49**	3.10	0.19	**0.02**	1.36	13.62
Varicocele	−1.65	2.28	−0.06	0.47	−6.15	2.84

*ANOVA test, P < 0.05*.

* *Model Predictors: (1) Age ≥ 45 years, (2) Obesity [BMI ≥ 30 kg/m^2^], (3) Sedentary lifestyle, (4) Current tobacco smoking ≥ 10 year-pack, (5) Daily alcohol consumption ≥ 3 doses according to WHO (10), (6) Marijuana smoker [≥1× per week/1 year], (7)Presence of clinical varicocele; SHBG, sex hormone-binding globulin; BMI, body mass index; WHO, World Health Organization. Bold values mean P < 0.05*.

**Table 8:** Coefficients of the multiple linear regression analysis model for dependent variable Semen volume.

Model predictor^*^	**Unstandardized coefficients**		**Standardized coefficient**	***P***	**95% confidence interval for beta**	
	**B**	**Stand error**	**Beta**		**Lower bound**	**Upper bound**
Semen volume	**3.74**	0.59	-	**0.00**	2.58	4.90
Age	**−0.03**	0.01	−0.19	**0.02**	−0.06	−0.00
Obesity	−0.47	0.29	−0.14	0.10	−1.04	0.09
Sedentary lifestyle	−0.20	0.23	−0.07	0.39	−0.65	0.26
Tobacco smoking	−0.56	0.47	−0.10	0.23	−1.49	0.36
Alcohol consumption	0.25	0.24	0.08	0.29	−0.22	0.72
Marijuana use	−0.15	0.35	−0.03	0.67	−0.85	0.55
Varicocele	**0.53**	0.26	0.17	**0.04**	0.02	1.04

*ANOVA test, P < 0.05*.

* *Model Predictors: (1) Age ≥ 45 years, (2) Obesity [BMI ≥ 30 kg/m^2^], (3) Sedentary lifestyle, (4) Current tobacco smoking ≥ 10 year-pack, (5) Daily alcohol consumption ≥ 3 doses according to WHO (10), (6) Marijuana smoker [≥1× per week/1 year], (7) Presence of clinical varicocele*.

*BMI, body mass index; WHO, World Health Organization. Bold values mean P < 0.05*.

## Discussion

The disturbing worldwide progressive decrease in semen quality over the last 40-years has multifactorial interconnected origins, including environmental, toxicological, behavioral, and lifestyle-related issues. Nevertheless, the continuous search for new clues or potential agents and close interactions among human and environmental factors has been considered an urgent task by government, institutions, and scientists (24). Our study demonstrates one previously unrevealed aspect of marijuana (*Cannabis sativa*) consumption with deleterious effects on a specific male cohort seeking fertility evaluation in a specialized andrology setting. It is widely accepted that marijuana cannabinoids compete with endo-cannabinoids to occupy cannabinoid receptors (CB1 and CB2) along the hypothalamus-pituitary-testicular axis (25). To this day, a large body of data emphasizes the role of cannabinoids in controlling reproduction by modifying gonadotropin release, fertility, and sexual behavior (26).

Our data bring forward a significant correlation between marijuana use and lower serum estradiol (E_2_) levels. The correlation also confirms a point between marijuana use and higher T/E2 ratios in the absence of any observed effect on testosterone levels. To date, conflicting data exist. While two studies based on the use of cellular models have reported that Δ9-tetrahydrocannabinol (THC), cannabinol (CBN), and cannabidiol (CBD), three major marijuana compounds, had neither pro- nor anti-estrogenic effect (27, 28), it was reported elsewhere that these same compounds might reduce E_2_ levels *via* their effect on the sex steroid metabolizing cytochrome P450 enzymes CYP3A and CYP1B (29, 30). However, these same studies reported that marijuana smoke condensate (MSC) showed both estrogenic and anti-estrogenic effects assumed to be mediated by other marijuana compounds (27). It was proposed that phenolic compounds could be responsible for the estrogenic effect (27), while polycyclic aromatic compounds (PAHs) might account for the anti-estrogenic effect most likely *via* the estrogen (ER) and the aryl hydrocarbon (AhR) receptor pathways (31–34). It was also proposed that MSC could also interfere with E_2_ biosynthesis via aromatase activity inhibition (28), which could agree with our observed increasing T/E_2_ ratios in marijuana users. Although THC, CBD, and CBN were not linked to any aromatase inhibiting activity (28), cannabidiorcol (CBD-C1), cannabitriol (CBT), and cannabiripsol (CBR), other biologically active marijuana cannabinoids have this effect (35). A pro-estrogenic action of marijuana consumption was also suggested at the clinical and epidemiological level since it was reported to be associated with gynecomastia (36).

Several habits and lifestyle factors influenced prolactin as its serum concentration was significantly modified by marijuana use, tobacco smoking, and sedentary in infertile men. Concerning the effects of marijuana use on serum prolactin concentration, it has been suggested that the brain cannabinoid receptor (CB-1R) is expressed close to hypothalamic dopaminergic receptors and that the administration of THC provokes an increase in dopamine (DA) release (37). Through negative feedback, DA influenced prolactin secretion by the anterior pituitary (37). The interfering effect of marijuana on DA receptors could explain our data. However, the literature is still controversial, as we reported an increase in serum prolactin levels in marijuana users, whereas some other studies observed no change (7, 11, 38) while others report a decrease (39). These divergent findings will have to be further investigated. Although prolactin's physiological importance for male reproduction remains uncertain, high levels of this hormone have been associated with male infertility, probably due to a direct deleterious effect on spermatogenesis and the inhibition of pulsatile gonadotropin release (40). Concerning the influence of a sedentary lifestyle on serum prolactin levels, it was reported that physical exercise was associated with greater prolactin secretion by the anterior pituitary (41). Although the correlation we found in our study between sedentary and serum prolactin levels were not extraordinarily strong, it appears to be positive and not negative as expected. Regardless, it remains within the physiological range, which may interrogate its actual relevance.

With tobacco smoking, we recorded a weak negative influence on prolactin serum levels. The literature exhibits conflicting data with some reports identical to ours (42, 43), but others reporting increased serum prolactin levels in tobacco users (44, 45). The adverse effect proposed is that nicotine could stimulate the central dopaminergic tonus, inhibiting prolactin release by the pituitary gland (42, 43). In an experimental study using rat pituitary cell-line, nicotine inhibited the prolactin gene expression (46).

Concerning SHBG, our data suggests regular marijuana consumption was positively associated with SHBG serum levels in infertile men. BMI is considered the primary determinant of SHBG circulating levels (47), mainly via hepatic visceral fat (48). Because of that, SHBG is classically seen as a biomarker for such conditions as metabolic syndrome, type 2 diabetes, and cardiovascular diseases (49). A Danish study concerning 1,215 young men reported similar data; however, once they controlled BMI and tobacco use, serum SHBG increase was no longer significant (8). It was recently suggested that SHBG testing could help evaluate infertile men, especially in the situation of hypogonadism (50), as SHBG binds androgens and estrogens and contribute to their bioavailability (51).

In the current study, we found that marijuana did not influence serum testosterone levels in infertile men. Once again, the literature reports conflicting data with results similar to ours (10–12, 52), in other cases, a decline (7) or even an increase (8) in serum testosterone level among cannabis users. Moreover, it is worthwhile to mention that semen quality and hormonal levels could be poor biomarkers for male fertility, while time-to-pregnancy (TTP) might be more representative to couples' fertility status. In this context, a recent large retrospective study revealed that marijuana use was not associated with TTP for men and women (53).

Nevertheless, we must report some limitations of our study. As is the case for cross-sectional studies, the causal inference of many questions may remain unclear. Retrospective studies are designed to evaluate pre-existing data; therefore may be subject to a reasonable and acceptable bias. Some complimentary data such as the duration of involuntary infertility, sexual activities, female partner age are missing. Concerning the applied heterogeneous definition of “marijuana use,” it is hard to distinguish more robust user (3–5 times a day) from those light consumers because temporal and quantitative relationships were often difficult to assess, and marijuana users tend to minimize or hide their habit, considered by many as relaxing and un-harmful. Furthermore, the lack of association between semen parameters' abnormalities and any of the habits studied may be justified by the lack of a matched control group of fertile “healthy” men.

Marijuana use is associated with hormonal imbalance in a cohort of infertile men, significantly lowering E_2_ serum levels while increasing prolactin serum concentrations. Further prospective studies with larger cohorts will be pertinent to corroborate our findings and improve the understanding of the underlying mechanisms that modify and regulate these perceivable endocrine changes. A warning notice should be addressed to marijuana consumers, particularly adolescents, young adults, and men in the reproductive age groups.

## Data Availability Statement

The raw data supporting the conclusions of this article will be made available by the authors, without undue reservation.

## Ethics Statement

The studies involving human participants were reviewed and approved by the Ethics Committee of the Faculty of Medicine of the University of São Paulo, Brazil. Written informed consent for participation was not required for this study in accordance with the national legislation and the institutional requirements.

## Author Contributions

TT, EC, and JH: conception and design of the study. II and GA: acquisition of data. TT, PS, JD, and JH: analysis and interpretation of data. TT, II, and GA: drafting the article. PS, JD, EC, and JH: revising it critically for intellectual content. All authors: final approval of the version to be submitted and agreement to be accountable for all aspects of the work in ensuring that questions related to the accuracy or integrity of any part of the work are appropriately investigated and resolved.

## Conflict of Interest

The authors declare that the research was conducted in the absence of any commercial or financial relationships that could be construed as a potential conflict of interest.

## Publisher's Note

All claims expressed in this article are solely those of the authors and do not necessarily represent those of their affiliated organizations, or those of the publisher, the editors and the reviewers. Any product that may be evaluated in this article, or claim that may be made by its manufacturer, is not guaranteed or endorsed by the publisher.
